# Value of lymph node ratio as a prognostic factor of recurrence in medullary thyroid cancer

**DOI:** 10.7717/peerj.15025

**Published:** 2023-03-13

**Authors:** Weijing Hao, Jingzhu Zhao, Fengli Guo, Pengfei Gu, Jinming Zhang, Dongmei Huang, Xianhui Ruan, Yu Zeng, Xiangqian Zheng, Ming Gao

**Affiliations:** 1 Department of Thyroid and Neck Tumor, Tianjin Medical University Cancer Institute and Hospital, National Clinical Research Center for Cancer, Key Laboratory of Cancer Prevention and Therapy, Tianjin’s Clinical Research Center for Cancer, Tianjin, China; 2 Department of Thyroid and Breast Surgery, Binzhou Medical University Hospital, Binzhou, Shandong, China; 3 Department of Thyroid and Breast Surgery, Tianjin Union Medical Center, Tianjin, China; 4 Tianjin Key Laboratory of General Surgery in Construction, Tianjin Union Medical Center, Tianjin, China

**Keywords:** Medullary thyroid cancer, Lymph node ratio, Metastatic lymph node, Resected lymph node, Disease-free survival, Overall survival

## Abstract

**Background and Objectives:**

The purpose of this study is to evaluate the relationship between lymph node status (the number of resected lymph nodes; the number of metastatic lymph nodes, LNM, and lymph node ratio, LNR) and biochemical recurrence, disease-free survival (DFS), as well as overall survival (OS) in medullary thyroid carcinoma (MTC).

**Methods:**

This study enrolled MTC patients at Tianjin Medical University Cancer Institute and Hospital between 2011 and 2019. We used Logistic regression analysis, Cox regression models and Kaplan-Meier test to identify risk factors influencing biochemical recurrence, DFS, and OS.

**Results:**

We identified 160 patients who satisfied the inclusion criteria from 2011 to 2019. We used ROC analysis to define the cut-off value of LNR with 0.24. Multifocality, preoperative calcitonin levels, pathologic N stage, resected lymph nodes, LNM, LNR, and the American Joint Committee on Cancer (AJCC) clinical stage were significant (*P* < 0.05) prognostic factors influencing biochemical cure. In univariable analyses, gross extrathyroidal extension, preoperative calcitonin levels, pathologic T classification, pathologic N stage, resected lymph nodes, LNM, LNR, AJCC clinical stage, and biochemical cure were significant (*P* < 0.05) factors of DFS. When the multivariable analysis was performed, LNR was identified as predictor of DFS (HR = 4.818, 95% CI [1.270–18.276]). Univariable Cox regression models reflected that tumor size, pathologic N stage, and LNR were predictor of OS. Furthermore, multivariable analysis manifested that LNR was predictor of OS (HR = 10.061, 95% CI [1.222–82.841]).

**Conclusions:**

This study illustrated that LNR was independent prognostic factor of DFS and OS in MTC. In addition, LNR influenced biochemical cure. Further investigations are needed to determine the optimal cut-off value for predicting prognosis.

## Introduction

Medullary thyroid carcinoma (MTC) is a rare C-cell-derived neuroendocrine malignancy. It accounts for 1–2% of all thyroid cancers in the United States. Most MTC cases (75%) are sporadic, while 25% are familial and associated with germ-line mutations (Erovic et al., 2012). MTC cells do not concentrate radioactive iodine and thyroid stimulating hormone insensitivity (Jin & Moley, 2016). Thus, surgical treatment is the mainstay of therapy. In MTC, lymph nodes metastasis can be detected in up to 75% of the patients (Moses et al., 2021; Polistena et al., 2017). In addition, 10-year survival rates for MTC are below 80%, and the cervical metastasis had significant influence on survival for MTC (Bhattacharyya, 2003; Brierley et al., 1996). The prognosis of MTC varied and disease related factors included age, gender, lymph node metastases, calcitonin levers, distant metastases, and response to initial treatment (Wells et al., 2015).

The current American Joint Committee on Cancer (AJCC) staging system for MTC categorizes lymph nodes (LN) status as N0 (no positive nodes), N1a (positive nodes in the central neck compartment), and N1b (positive nodes in the lateral neck) (Amin et al., 2017). It does not take into account the number of resected and positive nodes. Typically, the treatment of MTC involves routine central compartment dissection, and lateral neck dissection is recommended for patients with structural evidence of lateral compartment metastasis or with high preoperative calcitonin levels. Almost all patients will undergo some kind of lymph node dissection. Whether the lymph nodes status has any predictive value is not well illustrated.

The purpose of this study is to evaluate the relationship between lymph node status (the number of resected lymph nodes; the number of metastatic lymph nodes, LNM, and lymph node ratio, LNR) and biochemical recurrence, disease-free survival (DFS), as well as overall survival (OS). In addition, we test to investigate the optimal LNR cut-off value that best predicts the outcome.

## Materials and Methods

We retrospectively searched the databases for patients with MTC at Tianjin Medical University Cancer Institute and Hospital between 2011 and 2019. The present study was approved by the Institutional Review Board (bc2022191). Written informed consent was obtained from the patients.

All patients undergoing primary surgical treatment for MTC were included. Patients were excluded if they had pathologically positive resection margin, distant metastasis, or a history of thyroidectomy. Additionally, patients with a family history of MTC, a history of other malignancy, or incomplete data were not included.

Patient demographics, clinicopathologic factors, and survival outcomes were recorded. All the patients were operated on total thyroidectomy or hemithyroidectomy with central or both central and lateral compartment dissection considering preoperative imaging and calcitonin levels. Serum calcitonin was measured using the immunoradiometric assay. All the specimens in this study were analyzed by two or more dedicated head and neck pathologists. Recurrence was defined as the appearance of disease with pathology-confirmed local or distant disease detected by imaging scans 3 months after surgery. A biochemical cure was described as an abnormal preoperative calcitonin level declining within the reference range within 6 months after surgery. Patients’ follow-up primarily included neck ultrasound/CT and calcitonin levels.

We evaluated (1) the number of resected lymph nodes: 0 to 10, and greater than ten nodes; (2) LNM and (3) LNR, the number of metastatic lymph nodes divided by the number of resected lymph nodes. The nodal status was investigated in terms of its association with all the mentioned demographic, pathological, and prognostic variables. We used ROC analysis to define the cut-off value of LNR that best reflected prognosis.

Statistical analysis was performed using SPSS software (version 20.0; IBM, Chicago, IL, USA). Chi-squared analysis was used to compare frequencies between groups. Logistic regression analysis was used to identify risk factors influencing biochemical recurrence. Univariable and multivariable Cox regression models were applied to find risk factors influencing structural recurrence. Survival analysis was performed using Kaplan-Meier test. *P* < 0.05 was considered to indicate statistically significant differences.

## Results

### Baseline characteristics of the study population

We identified 160 patients who satisfied the inclusion criteria from 2011 to 2019. Demographic data are displayed in Table 1. The median age at the time of diagnosis was 52 years (14–73), and the majority of patients were female (90, 56.3%). The mean size of the largest tumor diameter was 1.79 cm, and 61 (38.1%) patients had an extrathyroidal extension. A total of 13 (8.1%) patients had bilateral tumors and 44 (27.5%) patients had multifocal tumors. Only central LN dissection was conducted in 77 (48.1%) patients. Meanwhile, central and lateral LN dissection was conducted in 82 (51.3%) patients. Approximately half of the patients had advanced stage MTC (stages III-IV, 59.4%). Positive lymph nodes were identified in 89 (55.6%) of cases. The median length of follow-up was 51 months (10–114 months). Structural recurrence was identified in 24 (15.0%) patients, and 12 (7.5%) patients died at the end of the study period. DFS and OS for the entire cohort were 83.1% and 91.3% at 5 years, respectively.

**Table 1:** Clinicopathologic characteristics of 160 medullary thyroid carcinoma patients.

Features	*N*	Percentage
Total	160	100 (%)
Age: years, median ± SD	52 ± 12.0	
Gender		
Male	70	43.8
Female	90	56.3
Tumor size (cm)		
≤2 cm	115	71.9
>2 cm	45	28.1
Multifocality		
Yes	44	27.5
No	116	72.5
Extrathyroidal extension		
Yes	61	38.1
No	99	61.9
Bilateral		
Yes	13	8.1
No	147	91.9
Pathologic T classification		
pT1	74	46.3
pT2	21	13.1
pT3	39	24.4
pT4	26	16.3
Pathologic N classification		
pN0	71	44.4
pN1a	22	13.8
pN1b	67	41.9
Resected lymph nodes: median (IQR)	14 (3–32)	
Metastasized lymph nodes: median (IQR)	1 (0–7)	
Lymph node ratio: median (IQR)	0.123 (0–0.377)	
AJCC clinical stage		
I	40	25.0
II	25	15.6
III	18	11.3
IV	77	48.1
Preoperative calcitonin: median (IQR)	521 (129–1,555)	
Lymph node dissection		
Only central LND	77	48.1
Central and lateral LND	82	51.3
Not done	1	0.6
Biochemical cure		
Yes	84	60.9
No	54	39.1
Unknown	22	13.8
Recurrence		
Yes	24	15.0
No	135	84.4
Unknown	1	0.6
Death		
Yes	12	7.5
No	147	91.9
Unknown	1	0.6
Follow-up duration: months, median (IQR)	51 (36–72)	

**Note:**

SD, standard deviation; IQR, interquartile range.

**Figure 1:**
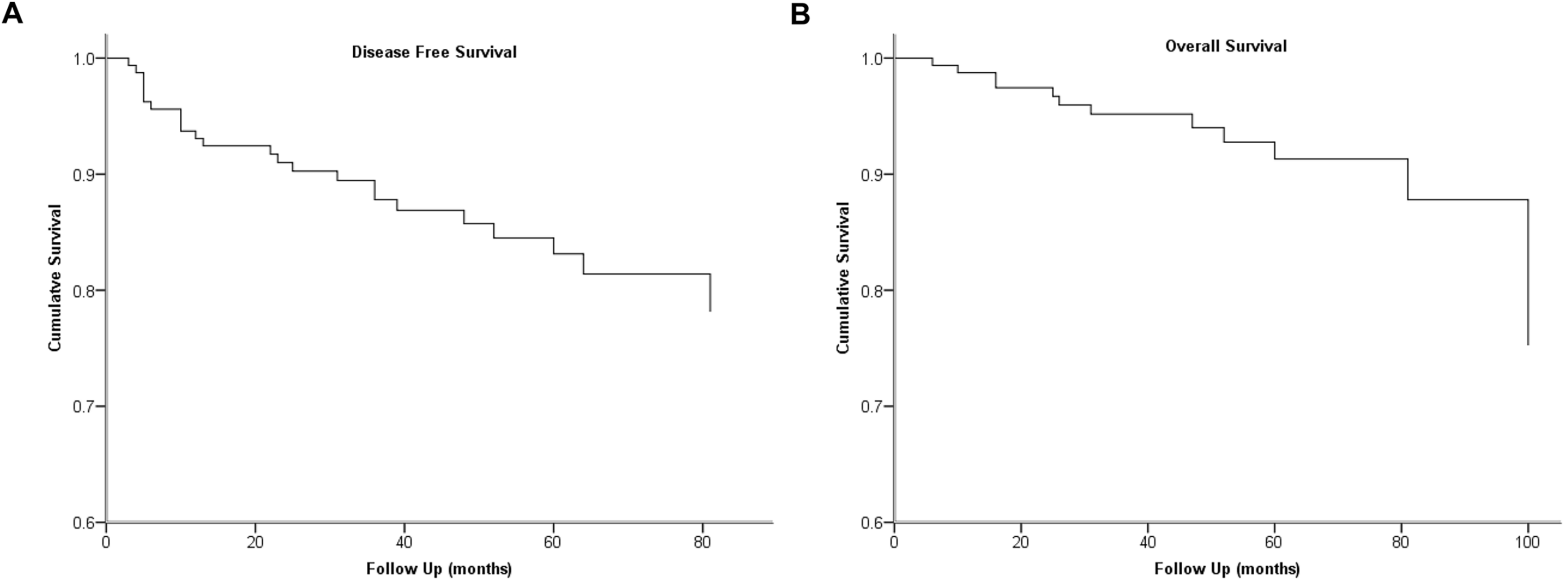
Kaplan-Meier survival plots present cumulative disease-free survival (A) and overall survival (B) of the cohort.

We used ROC analysis to define the cut-off value of LNR, and 0.24 was determined as the cut-off level with the highest predictive performance. The cumulative survivals of the cohort are shown in Fig. 1.

### Association of resected lymph nodes, LNM, LNR, and pathologic N classification with patient and tumor characteristics

The clinicopathologic characteristics of resected lymph nodes group, LNM group, LNR group, and pathologic N classification are shown in Tables 2 and 3. There were no significant differences in age, capsule invasion, and bilateral between groups, while prognostic factors varied.

**Table 2:** Clinicopathologic characteristics according to resected lymph nodes group and metastasized lymph nodes group.

Variables	Resected lymph nodes			Metastasized lymph nodes		
	≤10	>10	*P* value	≤10	>10	*P* value
Age (years)			0.118			0.636
≤50	30 (40.0)	44 (52.4)		63 (47.4)	11 (42.3)	
>50	45 (60.0)	40 (47.6)		70 (52.6)	15 (57.7)	
Gender			**0.036**			**0.013**
Male	26 (34.7)	43 (51.2)		52 (39.1)	17 (65.4)	
Female	49 (65.3)	41 (48.8)		81 (60.9)	9 (34.6)	
Tumor size (cm)			**0.004**			**0.027**
≤2 cm	62 (82.7)	52 (61.9)		100 (75.2)	14 (53.8)	
>2 cm	13 (17.3)	32 (38.1)		33 (24.8)	12 (46.2)	
Multifocality			**0.016**			**0.021**
Yes	14 (18.7)	30 (35.7)		32 (24.1)	12 (46.2)	
No	61 (81.3)	54 (64.3)		101 (75.9)	14 (53.8)	
Extrathyroidal extension			0.059			0.076
Yes	23 (30.7)	38 (45.2)		47 (35.3)	14 (53.8)	
No	52 (69.3)	46 (54.8)		86 (64.7)	12 (46.2)	
Bilateral			0.512			0.063
Yes	5 (6.7)	8 (9.5)		8 (6.0)	5 (19.2)	
No	70 (93.3)	76 (90.5)		125 (94.0)	21 (80.8)	
Preoperative calcitonin			**<0.001**			**<0.001**
≤300 ng/L	46 (63.0)	13 (16.2)		59 (46.1)	0 (0.0)	
>300 ng/L	27 (37.0)	67 (83.8)		69 (53.9)	25 (100.0)	
Pathologic T classification			**0.031**			0.057
T1/T2	51 (68.0)	43 (51.2)		83 (62.4)	11 (42.3)	
T3/T4	24 (32.0)	41 (48.8)		50 (37.6)	15 (57.7)	
Pathologic N classification			**<0.001**			**<0.001**
pN0	58 (77.3)	12 (14.3)		70 (52.6)	0 (0.0)	
pN1a	15(20.0)	7 (8.3)		21 (15.8)	1 (3.8)	
pN1b	2 (2.7)	65 (77.4)		42 (31.6)	25 (96.2)	
AJCC clinical stage			**<0.001**			**<0.001**
I/II	52 (69.3)	12 (14.3)		64 (48.1)	0 (0.0)	
III/IV	23 (30.7)	72 (85.7)		69 (51.9)	26 (100.0)	
Recurrence			**<0.001**			**0.025**
Yes	3 (4.0)	21 (25.3)		16 (12.0)	8 (32.0)	
No	72 (96.0)	62 (74.7)		117 (88.0)	17 (68.0)	
Death			**0.001**			0.217
Yes	0 (0.0)	12 (14.5)		8 (6.1)	4 (15.4)	
No	75 (100.0)	71 (85.5)		124 (93.9)	22 (84.6)	

**Note:**

The bold part of the *P* value represents *P* < 0.05.

**Table 3:** Clinicopathologic characteristics according to lymph node ratio group and pathologic N stage group.

Variables	LNR			Pathologic N stage group			
	≤0.24	>0.24	*P* value	pN0	pN1a	pN1b	*P* value
Age (years)			0.577				0.501
≤50	42 (50.6)	28 (45.9)		33 (46.5)	8 (36.4)	34 (50.7)	
>50	41 (49.4)	33 (54.1)		38 (53.5)	14 (63.6)	33 (49.3)	
Gender			**0.015**				**0.005**
Male	28 (33.7)	33 (54.1)		21 (29.6)	11 (50.0)	38 (56.7)	
Female	55 (66.3)	28 (45.9)		50 (70.4)	11 (50.0)	29 (43.3)	
Tumor size (cm)			0.733				0.163
≤2 cm	58 (69.9)	41 (67.2)		52 (73.2)	19 (86.4)	44 (65.7)	
>2 cm	25 (30.1)	20 (32.8)		19 (26.8)	3 (13.6)	23 (34.3)	
Multifocality			**<0.001**				**0.003**
Yes	13 (15.7)	26 (42.6)		12 (16.9)	4 (18.2)	28 (41.8)	
No	70 (84.3)	35 (57.4)		59 (83.1)	18 (81.8)	39 (58.2)	
Extrathyroidal extension			0.094				**0.012**
Yes	28 (33.7)	29 (47.5)		18 (25.4)	11 (50.0)	32 (47.8)	
No	55 (66.3)	32 (52.5)		53 (74.6)	11 (50.0)	35 (52.2)	
Bilateral			0.242				0.604
Yes	5 (6.0)	7 (11.5)		5 (7.0)	1 (4.5)	7 (10.4)	
No	78 (94.0)	54 (88.5)		66 (93.0)	21 (95.5)	60 (89.6)	
Preoperative calcitonin			**0.005**				**<0.001**
≤300 ng/L	38 (46.9)	14 (23.7)		37 (53.6)	15 (71.4)	8 (12.5)	
>300 ng/L	43 (53.1)	45 (76.3)		32 (46.4)	6 (28.6)	56 (87.5)	
Pathologic T classification			0.078				**0.006**
T1/T2	53 (63.9)	30 (49.2)		52 (73.2)	10 (45.5)	33 (49.3)	
T3/T4	30 (36.1)	31 (50.8)		19 (26.8)	12 (54.5)	34 (50.7)	
Pathologic N classification			**<0.001**				
pN0	55 (66.3)	0 (0.0)					
pN1a	5 (6.0)	17 (27.9)					
pN1b	23 (27.7)	44 (72.1)					
AJCC clinical stage			**<0.001**				**<0.001**
I/II	51 (61.4)	0 (0.0)		65 (91.5)	0 (0.0)	0 (0.0)	
III/IV	32 (38.6)	61 (100.0)		6 (8.5)	22 (100.0)	67 (100.0)	
Recurrence			**<0.001**				**<0.001**
Yes	4 (4.8)	19 (31.7)		2 (2.8)	3 (13.6)	19 (28.8)	
No	79 (95.2)	41 (68.3)		69 (97.2)	19 (86.4)	47 (71.2)	
Death			**<0.001**				**<0.001**
Yes	1 (1.2)	11 (18.0)		0 (0.0)	1 (4.5)	11 (16.4)	
No	81 (98.8)	50 (82.0)		70 (100.0)	21 (95.5)	56 (83.6)	

**Notes:**

LNR, lymph node ratio. The bold part of the *P* value represents *P* < 0.05.

### Prognostic factors influencing biochemical cure

In chi-squared analysis, multifocality, preoperative calcitonin levels, pathologic N stage, resected lymph nodes, LNM, LNR, and AJCC clinical stage were significant (*P* < 0.05) prognostic factors influencing biochemical cure (Table 4). While logistic regression analysis did not identify independent risk factors (Table 5).

**Table 4:** Clinicopathologic characteristics according to biochemical cure.

Variables	Biochemical cure			*P* value
	Total	Yes	No	
Age (years)				0.237
≤50	68 (49.3)	38 (45.2)	30 (55.6)	
>50	70 (50.7)	46 (54.8)	24 (44.4)	
Gender				0.215
Male	60 (43.5)	33 (39.3)	27 (50.0)	
Female	78 (56.5)	51 (60.7)	27 (50.0)	
Tumor size (cm)				0.198
≤2 cm	98 (71.0)	63 (75.0)	35 (64.8)	
>2 cm	40 (29.0)	21 (25.0)	19 (35.2)	
Multifocality				**0.030**
Yes	37 (26.8)	17 (20.2)	20 (37.0)	
No	101 (73.2)	67 (79.8)	34 (63.0)	
Extrathyroidal extension				0.242
Yes	53 (38.4)	29 (34.5)	24 (44.4)	
No	85 (61.6)	55 (65.5)	30 (55.6)	
Bilateral				0.082
Yes	10 (7.2)	3 (3.6)	7 (13.0)	
No	128 (92.8)	81 (96.4)	47 (87.0)	
Pathologic T classification				0.096
pT1/T2	81 (58.7)	54 (64.3)	27 (50.0)	
pT3/T4	57 (41.3)	30 (35.7)	27 (50.0)	
Preoperative calcitonin				**0.001**
≤300 ng/L	51 (37.0)	40 (47.6)	11 (20.4)	
>300 ng/L	87 (63.0)	44 (52.4)	43 (79.6)	
Pathologic N classification				**<0.001**
pN0	60 (43.5)	51 (60.7)	9 (16.7)	
pN1a	21 (15.2)	12 (14.3)	9 (16.7)	
pN1b	57 (41.3)	21 (25.0)	36 (66.7)	
Resected lymph nodes				**<0.001**
≤10	65 (47.1)	51 (60.7)	14 (25.9)	
>10	73 (52.9)	33 (39.3)	40 (74.1)	
Metastasized lymph nodes				**<0.001**
≤10	118 (85.5)	80 (95.2)	38 (70.4)	
>10	20 (14.5)	4 (4.8)	16 (29.6)	
Lymph node ratio				**<0.001**
≤0.24	73 (57.9)	58 (76.3)	15 (30.0)	
>0.24	53 (42.1)	18 (23.7)	35 (70.0)	
AJCC clinical stage				**<0.001**
I/II	54 (39.1)	48 (57.1)	6 (11.1)	
III/IV	84 (60.9)	36 (42.9)	48 (88.9)	
Recurrence				**0.002**
Yes	20 (14.5)	6 (7.1)	14 (25.9)	
No	118 (85.5)	78 (92.9)	40 (74.1)	
Death				0.711
Yes	8 (5.8)	4 (4.8)	4 (7.5)	
No	129 (94.2)	80 (95.2)	49 (92.5)	

**Note:**

The bold part of the *P* value represents *P* < 0.05.

**Table 5:** The logistic regression analysis between biochemical cure and clinicopathological features.

	OR	95% CI	*P* value
Multifocality	1.861	[0.667–5.191]	0.235
Preoperative calcitonin ≤300 ng/L	1.969	[0.626–6.191]	0.246
Pathologic N classification			0.983
pN1a	0.954	[0.061–13.924]	0.954
pN1b	0.813	[0.046–14.464]	0.888
Resected lymph nodes ≤10	1.998	[0.481–8.302]	0.341
Metastasized lymph nodes ≤10	1.629	[0.371–7.150]	0.518
Lymph node ratio ≤0.24	2.532	[0.730–8.787]	0.143
AJCC clinical stage, III/IV stages	4.965	[0.367–67.265]	0.228

**Note:**

OR, odds ratio; CI, confidence interval.

### Prognostic factors influencing disease-free survival and overall survival

**Figure 2:**
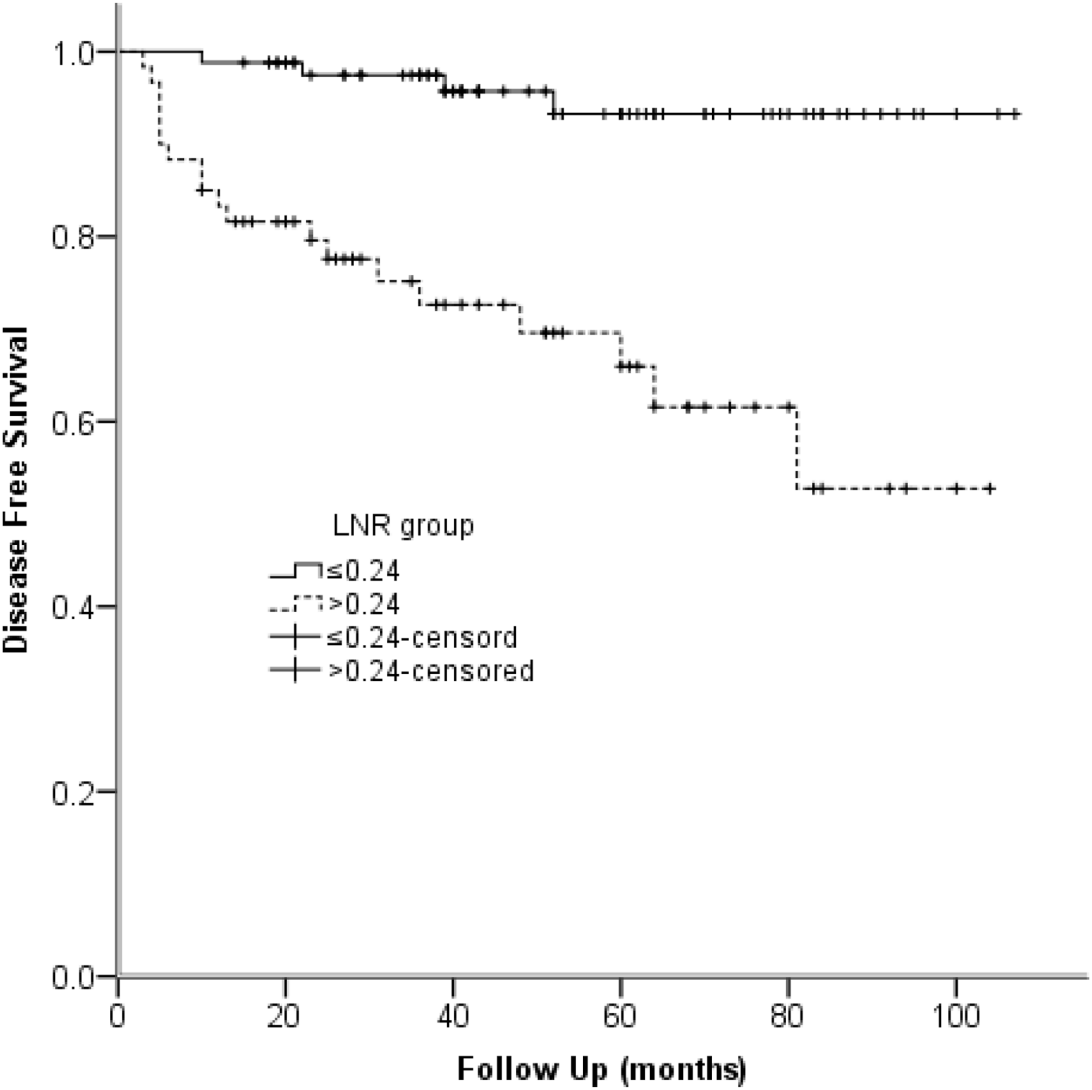
Kaplan-Meier survival plots present disease-free survival stratified by LNR group. Note: *P* < 0.001.

We used univariable and multivariable Cox regression models to identify the clinical characteristics affecting structural recurrence. In univariable analyses, gross extrathyroidal extension, preoperative calcitonin levels, pathologic T classification, pathologic N stage, resected lymph nodes, LNM, LNR, AJCC clinical stage, and biochemical cure were significant (*P* < 0.05) factors of DFS. When the multivariable analysis was performed based on the meaningful variables selected from univariate regression, LNR was identified as predictor of DFS (HR = 4.818, 95% CI [1.270–18.276]; *P* = 0.021) (Table 6). The Kaplan-Meier plot of DFS for LNR is provided in Fig. 2.

**Table 6:** Univariate and multivariate Cox regression models for predicting disease-free survival.

Variables	Univariate analysis			Multivariate analysis		
	HR	95% CI	*P* value	HR	95% CI	*P* value
Age ≤50 years	0.471	[0.206–1.077]	0.074			
Gender, male	0.661	[0.296–1.478]	0.313			
Tumor size >2 cm	2.175	[0.964–4.907]	0.061			
Multifocality	2.153	[0.953–4.865]	0.065			
Extrathyroidal extension	3.146	[1.345–7.359]	**0.008**	1.116	[0.138–9.001]	0.918
Bilateral	0.779	[0.183–3.324]	0.736			
Preoperative calcitonin ≤300 ng/L	8.120	[1.898–34.743]	**0.005**	1.728	[0.257–11.616]	0.574
Pathologic T classification, pT3/T4	3.531	[1.463–8.519]	**0.005**	1.664	[0.195–14.178]	0.641
Pathologic N classification, pN0/N1a	6.075	[2.267–16.281]	**<0.001**	1.482	[0.152–14.483]	0.735
Resected lymph nodes ≤10	7.412	[2.208–24.874]	**0.001**	3.242	[0.356–29.546]	0.297
Metastasized lymph nodes ≤10	3.516	[1.491–8.290]	**0.004**	0.469	[0.153–1.434]	0.184
Lymph node ratio ≤0.24	7.971	[2.708–23.463]	**<0.001**	4.818	[1.270–18.276]	**0.021**
AJCC clinical stage, III/IV stages	16.676	[2.251–123.546]	**0.006**	1.128	[0.071–17.965]	0.932
Biochemical cure	4.397	[1.686–11.468]	**0.002**	1.486	[0.512–4.316]	0.467

**Notes:**

HR, hazard ratio; CI, confidence interval. The bold part of the *P* value represents *P* < 0.05.

**Figure 3:**
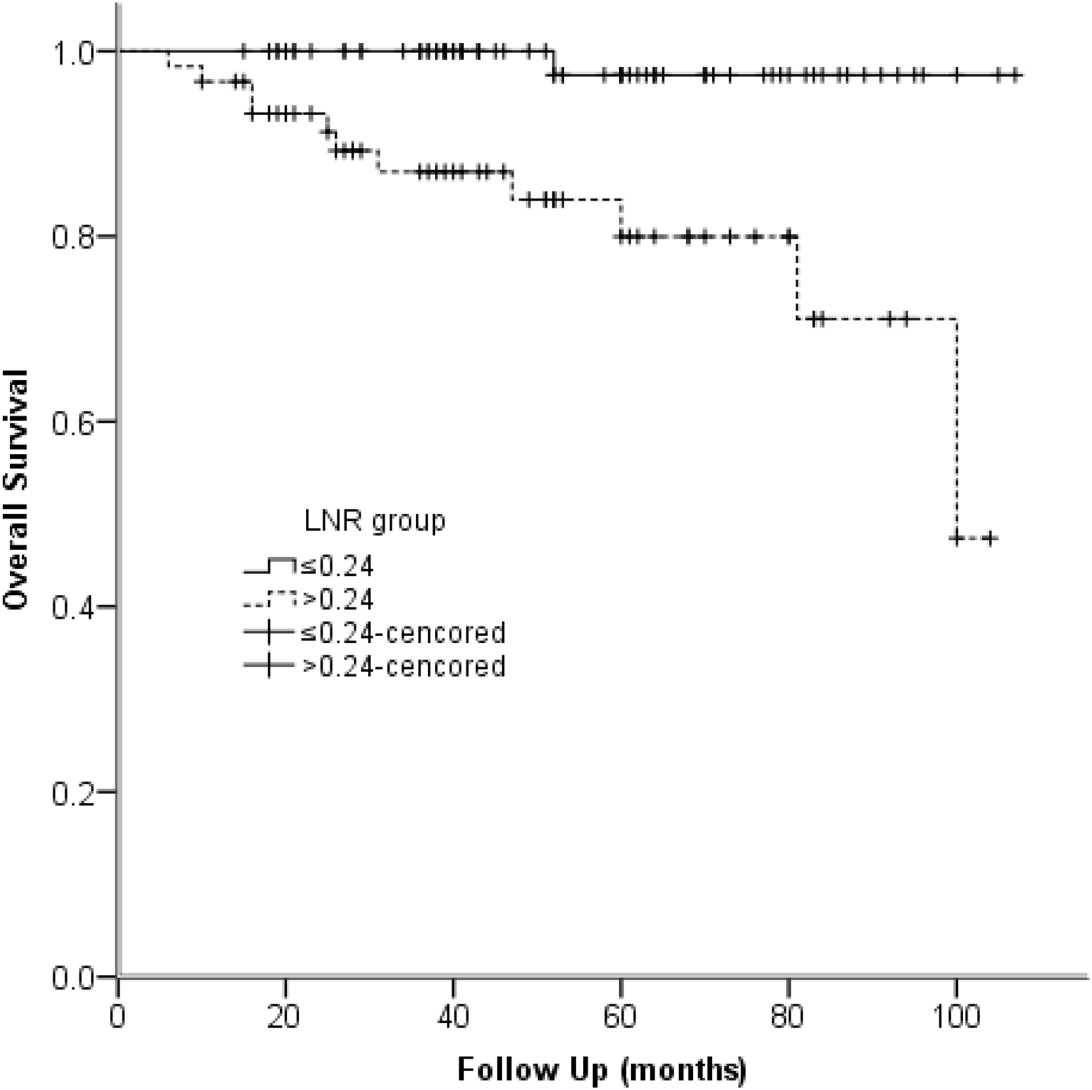
Kaplan–Meier survival plots present overall survival stratified by LNR group. Note: *P* < 0.001.

Univariable Cox regression models reflected that tumor size, pathologic N stage, and LNR were identified as predictors of OS. Furthermore, multivariable analysis manifested that LNR was predictor of OS (HR = 10.061, 95% CI [1.222–82.841]) (Table 7). The Kaplan-Meier plot of OS for LNR is provided in Fig. 3.

**Table 7:** Univariate and multivariate Cox regression models for predicting overall survival.

Variables	Univariate analysis			Multivariate analysis		
	HR	95% CI	*P* value	HR	95% CI	*P* value
Age ≤50 years	0.785	[0.252–2.439]	0.675			
Gender, male	0.606	[0.191–1.919]	0.394			
Tumor size >2 cm	4.385	[1.375–13.989]	**0.012**	2.847	[0.870–9.312]	0.084
Multifocality	1.536	[0.458–5.146]	0.487			
Extrathyroidal extension	2.965	[0.887–9.908]	0.077			
Bilateral	0.649	[0.083–5.095]	0.681			
Preoperative calcitonin ≤300 ng/L	6.943	[0.881–54.724]	0.066			
Pathologic T classification, pT3/T4	2.801	[0.838–9.361]	0.094			
Pathologic N classification, pN0/N1a	14.947	[1.922–116.264]	**0.010**	4.521	[0.516–39.627]	0.173
Resected lymph nodes ≤10	64.123	[0.597–6890.514]	0.081			
Metastasized lymph nodes ≤10	3.251	[0.971–10.884]	0.056			
Lymph node ratio ≤0.24	15.994	[2.063–124.023]	**0.008**	10.061	[1.222–82.841]	**0.032**
AJCC clinical stage, III/IV stages	43.503	[0.332–5694.725]	0.129			
Biochemical cure	1.870	[0.467–7.488]	0.376			

**Notes:**

HR, hazard ratio; CI, confidence interval. The bold part of the *P* value represents *P* < 0.05.

## Discussion

Previous studies have indicated that resected lymph nodes number, metastatic lymph nodes number, and ratio of metastatic lymph nodes to the total number of lymph nodes resected tended to be associated with survival outcomes in MTC patients (Leggett et al., 2008; Machens & Dralle, 2013; Moses et al., 2021). Whereas the current AJCC TNM classifications for MTC categorizes lymph node metastases, not by number but location of metastatic nodes. Patients belonging to the same pathologic N stage do not have equal disease burden. Thus, the American Thyroid Association Task Force suggested that lymph node status should be incorporated into the AJCC staging systems for predicting outcomes and planning long-term follow-up of MTC patients (Wells et al., 2015).

The present retrospective study aimed to investigate the role of resected lymph nodes, LNM, and LNR for predicting biochemical and structure recurrence in MTC. Multifocality, preoperative calcitonin levels, pathologic N stage, resected lymph nodes, LNM, LNR, and AJCC clinical stage were significant prognostic factors influencing biochemical cure. In addition, we found LNR was an independent prognostic factor of DFS and OS.

The current guidelines for MTC lack a specific lymph node number to guarantee the adequacy of the lymph node dissection and cannot reflect the effects of surgery. Thus, the number of resected nodes, LNM as well as LNR might provide more meaningful prognostic information for MTC patients who undergo surgery. In a previous study that enrolled 2,627 MTC patients, the number of positive nodes was divided into four groups, 0, 1 to 10, 11 to 20, and greater than 20 positive nodes. It manifested patients with 11 to 20 positive central lymph nodes had significantly worse survival than patients with 1 to 10 (Moses et al., 2021). Likewise, Machens & Dralle (2013) came to the same conclusion. Consequently, we classified both resected and metastatic lymph nodes into two groups, 0 to 10, and greater than ten nodes considering our fewer samples than the researchers above.

In our study, the chi-squared analysis indicated that resected lymph nodes, LNM, and LNR were significant prognostic factors influencing biochemical cure (Table 4). While, logistic regression analysis did not get positive results (Table 5). More samples may be available to get more profound effects. Nevertheless, multiple studies have found that postoperative serum calcitonin is a significant prognostic factor (Grozinsky-Glasberg et al., 2007; Yang et al., 2015). Therefore, the status of nodes may also be used in combination with postoperative calcitonin levels to predict patients’ prognosis (Yip et al., 2011).

To some extent, the number of resected and metastatic lymph nodes relies on both surgery and pathologic processing. By contrast, the LNR, which is the number of metastatic lymph nodes divided by the number of resected lymph nodes, maybe a better independent prognostic factor regardless of the personal skill level. We used ROC analysis to define the cut-off value of LNR. Finally, we chose 0.24 to differentiate the high- and low-risk groups for structural recurrence. In univariable studies, pathologic N stage, resected lymph nodes, LNM, and LNR were significant (*P* < 0.05) prognostic factors of DFS (Table 6). Furthermore, multivariable analysis manifested LNR was an independent predictor of DFS (HR = 4.818, 95% CI [1.270–18.276]; *P* = 0.021). Figure 2 demonstrates DFS between high-risk and low-risk series. Moreover, 5-year DFS was 93.2% and 65.9% in different risk groups. Rozenblat et al. (2020) and Jiang et al. (2017) reached an agreement with our study. By contrast, several previous studies have different LNR cut-off values varied from 0.10 to 0.50 (Kim et al., 2021; Qu et al., 2016; Rozenblat et al., 2020). Therefore, studies with a more extended follow-up period and a larger population are needed to determine the optimal cut-off value of LNR. What’s more, LNR is calculated right after the initial treatment of surgery. And previous studies focusing on other tumors have found that LNR can serve as a reliable prognostic factor (Mansour et al., 2018; Mizrachi et al., 2013).

Univariable Cox regression models demonstrated that LNR, pathologic N classification, and tumor size were predictors of OS (*P* < 0.05) (Table 7). Additionally, multivariable analysis manifested that LNR was predictor of OS (HR = 10.061, 95% CI [1.222–82.841]). Jiang et al. (2017) also stated that LNR was significantly associated with OS. The Kaplan-Meier plot illustrated that OS in LNR high-risk group was 80.0% at 5 years and 97.4% in the low-risk group (Fig. 3).

The present study found that LNR had the strongest association with DFS and OS, which is consistent with the previous studies. Meanwhile, LNR was a predictor of biochemical cure. These findings may help make up a revised staging classification that incorporates the status of nodes.

The limitation of this study is its retrospective design at a single center. Additionally, we did not include all patients with MTC, instead limiting our survey to those sporadic MTC patients. Finally, more patients and more extended follow-up periods are needed.

## Conclusion

In conclusion, this study illustrated that LNR was independent prognostic factor of DFS and OS in MTC. In addition, LNR influenced biochemical cure. Further investigations are needed to determine the optimal cut-off value for predicting prognosis.

## Supplemental Information

10.7717/peerj.15025/supp-1 Supplemental Information 1 Raw data.

## Acronyms

MTC: Medullary thyroid carcinoma
AJCC: American Joint Committee on Cancer
LNM: Metastatic lymph nodes
LNR: Lymph node ratio
DFS: Disease-free survival
OS: Overall survival

## Additional Information and Declarations

### Data Availability

The following information was supplied regarding data availability: The raw measurements are available in the Supplemental File.
